# Acquisition of genome information from single-celled unculturable organisms (radiolaria) by exploiting genome profiling (GP)

**DOI:** 10.1186/1471-2164-7-135

**Published:** 2006-06-02

**Authors:** Mariko Kouduka, Atsushi Matuoka, Koichi Nishigaki

**Affiliations:** 1 Department of Functional Materials Science, Saitama University, Saitama, Japan; 2 Department of Geology Historical Geology, Niigata University, Niigata, Japan

## Abstract

**Background:**

There is no effective method to obtain genome information from single-celled unculturable organisms such as radiolarians. Even worse, such organisms are often very difficult to collect. Sequence analysis of 18S rDNA has been carried out, but obtaining the data has been difficult and it has provided a rather limited amount of genome information. In this paper, we have developed a method which provides a sufficient amount of data from an unculturable organism. The effectiveness of this method was demonstrated by applying it to the provisional classification of a set of unculturable organisms (radiolarians).

**Results:**

Dendrogram was drawn regarding the single-celled unculturable species based on the similarity score termed PaSS, offering a consistent result with the conventional taxonomy of them built up based on phenotypes. This fact has shown that genome profiling-based technology developed here can obtain genome information being sufficient for identifying and classifying species from a single-celled organism.

**Conclusion:**

Since this method is so simple, general, and yet powerful, it can be applied to various organisms and cells, especially single-celled, uncluturable ones, for their genome analysis.

## Background

Even in the post-genomic era, genome information is often difficult to obtain. This is especially true for organisms that are difficult to collect due to the extremes of their living environment. Radiolaria are protozoa used in the study of geology and environmental and ecological sciences because they leave a fossil of hard skeletal material that can provide a record of more than 500 million years. Studies on Radiolaria have progressed since the early work by Haeckel [1,2], which was based on phenotypic analysis. The diverse shapes of their skeletons have been useful characteristics for the identification of species. However, there is a need to obtain genomic information about these organisms in order to more fully understand their diversity and phylogeny. Recently, the gene encoding a small subunit of ribosomal RNA (18S rDNA) has been used for classifying species of Radiolaria, which are taxonomically controversial [3-5]. This is by no means an easy task not only because of the difficulty in collecting samples, but also the additional complication that each organism is composed of only a single cell, and methods for culturing Radiolaria in the laboratory are not available. This means that only a single set of genomic DNA molecules can be obtained for experiments such as PCR. Species identification and/or classification from a single cell has an ultimate value for various purposes: genome analysis of precious specimens using only a minute fraction of the available sample or tracing alterations in the genome of somatic cells (e.g., differences between the genomes of the cells from the brain and heart). It will be also useful for settling controversial classification problems regarding Radiolarian [6]. The sequence information obtained from 18 S rDNA is often insufficient to identify and classify species in detail, wanting a method which can provide a more amount of information. This challenge is addressed in this paper: the authors developed a general way to obtain genome information from a single cell. For this purpose, a triple-session of random PCR was successfully employed. The experimental results obtained by genome profiling (GP) [7-10] of a group of Radiolaria gave consistent data from a taxonomical viewpoint. The authenticity of the results, including sequence analysis data, is extensively discussed.

## Results and discussion

### Experimental materials and genome profiling

**Figure 1:**
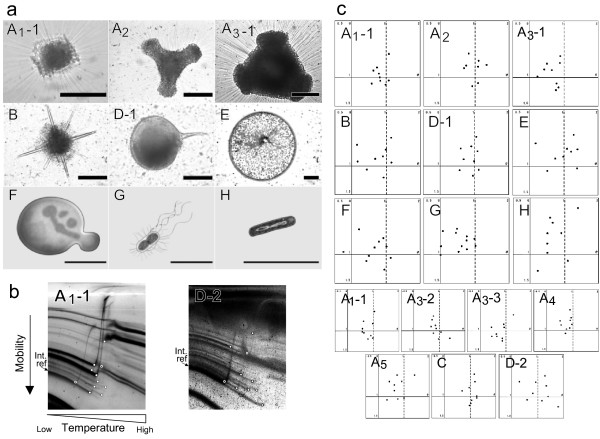
**Phenotype and genotype (*spiddos*)**. Photomicrographs A_1_~E are of freshly collected organisms used in this study. Photomicrographs F~H were taken from published books [22, 23]. a) Photomicrographs of Rhizaria (A_1_~D_2_), diatom (E), fungi (F) and bacteria (G & H). The scale bars correspond to 100 Âµm (thick line) and 5 μm (thin line). b) Genome profiles (GP) of radiolarian-derived DNA. The directions of migration and temperature gradient are shown. The DNA band of the internal reference for each GP is shown. The small white circles indicate the featuring points termed *species identification dots (spiddos)*. DNAs were fluorescently-labeled (left) or silver-stained (right). c) Normalized *spiddos*, which represent the genotype for each organism. Panels of A_1 _to H correspond to the same organisms as in Fig. 1a. The remaining 8 panels without pictures contain GPs for the organisms examined in this paper. GPs of A_1_-1 and D-2 are shown in Fig. 1b. The original data (i.e., genome profiles) for *spiddos *of panels Fig. 1c-F and -G were published in refs 13 and 19, respectively, newly processed to *spiddos *here. The sample foe the panel Fig. 1c-H is newly reported.

Three sessions of random PCR (70 cycles in all) enabled us to obtain DNA fragments from single-celled organisms. The DNA fragments were subjected to micro-TGGE [11], providing genome profiles as shown in Fig. 1b. In this series of experiments, currently unculturable and difficult-to-collect species of Haeckel's Radiolaria and related organisms (C and E in Table 1) were collected in the field and examined (Fig. 1). The genome profiles of these organisms were processed to generate species identification dots (spiddos) as shown in Fig. 1c. Since these dots were obtained after standardization and normalization by utilizing the internal reference (see Materials and Methods), the coordinates of these spiddos can be regarded as sufficiently reproducible, intrinsic properties of each genome without the influence of environmental factors [12,13]. The visual pattern of spiddos of an organism on the plane tells us roughly which type of family it belongs to. On the other hand, phenotypes of organisms such as those shown in Fig. 1a provide abundant information about the environment that must have affected the formation of the detailed shape and size. Therefore, spiddos can be used to assign the species and measure the difference (distance) between two organisms at the genome level.

### PaSS and clustering analyses

**Figure 2:**
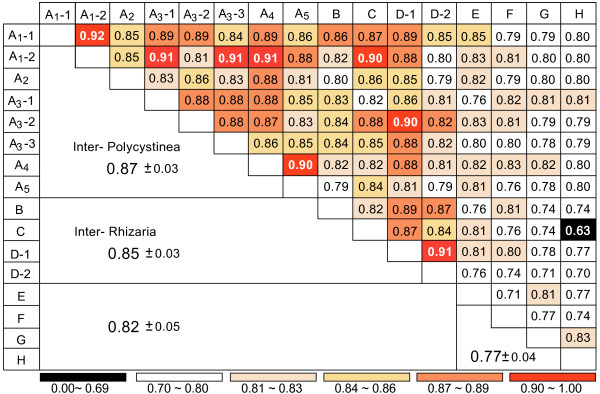
**Genome similarities between single-celled organisms measured by the pattern similarity score (PaSS)**. Alphabetical letters represent the species described in Table 1. The area to the right of the diagonal accommodates PaSS values between a pair of species (for an exact match, PaSS = 1), while the area to the left of the diagonal contains the mean of the PaSS values located in the area.

Quantitative analysis of spiddos has been established as PaSS (pattern similarity score) analysis [13]. In preceding studies, the reproducibility of this method has been thoroughly examined and established to be within 1% of errors as PaSS values for a moderately trained person of this technology, which were obtained by measuring the self-PaSS, i.e., the PaSS value for the same genome (operationally, this is obtained based on two genome profiles through two independent trials beginning with the same culture (clone) or with the same tissue of an organism). In other words, the same sample usually provides the PaSS value of 0.986 ± 0.003, if compared with each other [13]. As shown in Fig. 2, PaSS values for each pair of Polycystinea are significantly larger (c.f., PaSS value of 1 indicates a complete match and 0.435 ± 0.091 for non-related two genomes [13]) in comparison to those of the other pairs. In contrast, pairs composed of a radiolarian and a bacterium or a bacterium and a fungus gave significantly lower PaSS values, which is clearly shown in the lower left triangle of Fig. 2. To evaluate the PaSS values obtained here, we can refer to a list of PaSS made for all organisms so far examined (Table 2). Although these values were statistically obtained based on GP data of more than 100 species and they may change depending on the number and the species of organisms analyzed, they can be used with a finite reliability as a reference to estimate the difference between two genomes at a particular level of taxon. According to Table 2, the values presented in Fig. 2 (~0.85) seem relatively high (almost the level of genus), and this result may indicate the degree of similarity of genomes in Haeckel's Radiolaria.

**Figure 3:**
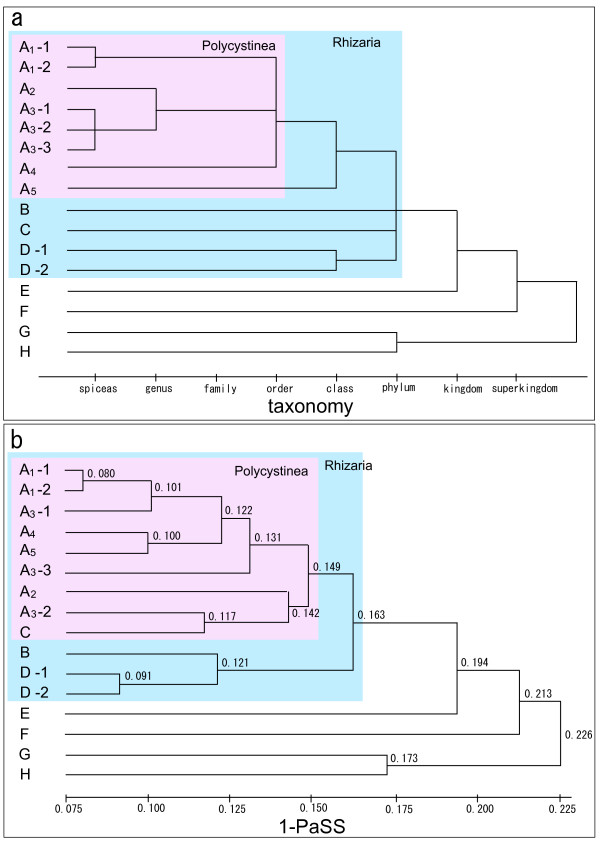
**Phylogenetic trees of single-celled organisms**. a) Phenotype based dendrogram. Taxonomical classification (abscissa) is followed after that supported by Pub Med [24]. b) Genome distance-based dendrogram. Genome distance is defined as (1-PaSS). The group average method (GAM) was used [14]. Haeckel's Radiolaria is A, B, C.

Phenotype tree is drawn in Fig. 3a. On the other hand, we employed a clustering analysis (group average method)[14] to the PaSS scores and obtained the results shown in Fig. 3b. A reasonable result was obtained at a distance for clustering of 0.149 where all of the members of Polycystinea (A_1_~A_5_) were integrated in the same cluster though it includes Foraminiferida (C) as a unique stringer, and there are some rearrangements in branches among the species of Polycystinea. At the same time, mutually distant species such as diatom, bacteria, and fungus (E~H) were separate. At the distance of 0.163, all of the Rhizaria (A_1_~A_5_, B~D) were combined into a cluster while the other mutually-distant species (E~H) were still isolated, conforming to the concept of traditional taxonomy. If we consider that the classification of Polycystinea is under construction and there is generally an experimental error in the PaSS values of approximately 1 % [13] and that Some Radiolaria, such as the groups Polycystinea A_1 _and A_3 _are well known to have symbiotic algae within the cell [15], while other Polycystinea (A_2_, A_4_, A_5_) do not, the apparent minor differences may be insignificant (in other words, beyond the resolution of the current approach) and as a whole, the entire clustering seems rational. Therefore, these results strongly indicate that the method applied here, i.e., random PCR-based genome profiling, could provide the sufficient amount of genomic information from a single cell. The GP method supported the classical Haeckel's view of Radiolaria as monophyletic group, and remained in opposition to the polyphyly of Radiolaria suggested by rRNA and actin phylogenies [6,16] although the results obtained here are provisional.

### Reliability tested by sequencing

Since there are no published genome sequences for any radiolarians or related organisms, the results obtained here must be critically evaluated based on other available information. Currently, the most common approach is to sequence the 18S rDNA; however, it is very difficult to carry out both GP and 18S rDNA sequencing experiments with single-celled and unculturable organisms (only a single copy of each DNA molecule is available). However, anyhow, we tried to amplify the 18S rDNA genes with specific PCR primers (see Materials and Methods) using the template DNA used for GP as the template (which, naturally, contations random PCR products also). We were not always able to amplify the target molecule (18S rDNA). This may be due to a stochastic or inevitable loss of the template DNA when dealing with a single copy. Though we can expect least to obtain 18S rDNA from the mixture of DNAs obtains after genome profiling, DNA molecules amplified using the primers designed for 18S rDNA from the genome of one sample (*Dictyocoryne profunda*) happened to have around the expected molecular size (200 bp) and were sequenced. After searching the database (DDBJ) with FASTA, the sequence obtained was found not to be the same as any of those reported to date (data not shown). Sequences currently in the database included *Didymocyrtis tetrathalamus*, *Dictyocoryne profunda*, and *Dictyocoryne truncatum*. In addition to these, Acantharea (11 entries), Phaeodarea (3 entries), Polycystinea (20 entries) have been reported. The results of our analysis are as follows: i) the sequence obtained was so novel that it had not been reported previously, ii) it had the highest similarity (66.7 %) with partial 18S rDNA of a protozoan, Euglena spathirhyncha, iii) none of the small subunit ribosomal DNA sequences of organisms that might be expected as contaminants, such as algae, planktonic organisms, fungi or bacteria were highly ranked within the top 200 sequences. Therefore, although the result could not be reproducibly tested, as the same genome cannot be obtained again, it is very plausible that the DNA sequence we obtained and used for GP analysis was that of a Radiolarian 18S rDNA that had never been sequenced. At the same time, we also sequenced the random PCR products of samples A_2_, A_3_-1, 2, B, D-1 and others (70 reads in total, 305 nucleotides on average; data not shown) and found that: i) the sequences obtained were quite novel with no registered sequences having more than 70 % similarity except one *E. coli *sequence (99 % similarity) and two algae sequences (81 % and 77 % similarities), and ii) according to the FASTA search, sequences with the highest similarity with the query sequences were not those of probable contaminants but those of protozoa, which are related to Radiolaria. According to the first finding, an accidental contamination of E. coli DNA must have happened during the experiment. It may also mean that some of our samples were contaminated with algae. However, since they are not major representatives in the sequencing results for 70 clones, they must not have contributed significantly to the spiddos generated in the GP experiments. It is already known that a minor population of DNAs can not be represented in GP unless they have exceptionally strong binding sites for a primer or highly repetitive sequences that can be amplified by random PCR. Therefore, these findings provide supporting evidence that the random PCR products were derived from the genomes of Radiolaria.

In conclusion, the results presented here, including the data indicating that GPs of Radiolarians provided a taxonomically-consistent clustering result (Figs. 2 and 3), indicate that genome profiles for Radiolarians can be obtained from a single cell. Here, we need to recall the fact that GPs obtained here may consist of composite genomes, e.g., those of host and parasitic/symbiotic organisms which can not be observed by the conventional microscopic techniques (though employing more sophisticated technologies such as SEM will make it possible). In other words, some of GPs reported here may represent a metagenome, or 'an ensemble of genomes' (originally, GP was developed based on the idea that the genome is the whole ensemble of DNAs contained in a cell [17]). If so, it is reasonable to register such GPs as being representative of Radiolaria since we cannot separate the symbiotic genomes effectively, and this type of partnership is often permanent. We think this fact dose not devaluate the methodology established here.

## Conclusion

In this paper we developed a potent method to obtain genome information from unculturable and single-celled organisms. This was demonstrated by the experiments applied to radiolarians sampled in the field. Final confirmation can only be obtained by the repeating similar experiments using other fresh experimental materials. Therefore, the classification reported here should be considered to be rather tentative, but the GP method shows promise for solving controversial classification problems. We believe this is the first success in which the genomes of unculturable, single-celled organisms were treated quantitatively and systematically to extract genomic information for comparison.

## Methods

### DNA of radiolarians and the other organisms

The samples of protozoa were collected from surface seawater during 2003–2005. Protozoa, such as species belonging to Phaeodaria and Acantharia, Foraminifera, and Diatomophyceae were obtained from the shores off of Sado Island, Japan. Radiolarians, including *Dictyocoryne truncatum*, *Dictyocoryne profunda*, *Didymocyrtis tetrathalamus*, *Spongaster tetras*, *Dictyocoryne truncatum*, *Eucyrtidium hexasticum*, *Hymeniastrum euclidis *were from the shores off of Okinawa Island, Japan. The collected organisms are listed in Table 1 with their taxonomical classification and sampling date and location. Photomicrographs of some of the organisms are shown in Fig. 1a. The plankton-net collected microbes were dissolved in sterile water. Then we prepared each organism as a single cell by sucking it together with a small aliquot(less than 1 μl including a cell) under the microscopic view without contaminating the other visible organisms and diluted it with pure alcohol of more than 1 ml. An aliquot (1 μl) of this solution which contains the cell was used for PCR. Thus, the sample cells used here were microscopically pure but it can not rule out the possibility of being contaminated with intracellular organisms (symbionts) or externally invisible virus-like organisms (though its probability is less than a millionth of that of finding in the original sea water due to the repeated dilution). The other organisms dealt here (*Saccharomyces cerevisiae*, *Escherichia coli*, and *Bacillus subtilis*) were reported previously [10,13].

### DNA preparation

Each sample was purified by picking up a single-cell in an aliquot. Preparation of DNA was carried out by the alkaline extraction method [18]. Briefly, the procedures adopted here are as follows: 1) An aliquot containing a single cell which had been identified by phenotype under the microscope was transferred into an Eppendorf tube; 2) After adding 3 μl of 0.5 M NaOH, the sample solution was incubated at 94°C for 5 min and then at 64°C for 60 min; 3) the sample solution was neutralized with 5 μl of 200 mM Tris-HCl (pH 8.0) buffer, and incubated at 65°C.

### Genome profiling (GP)

**Figure 4:**
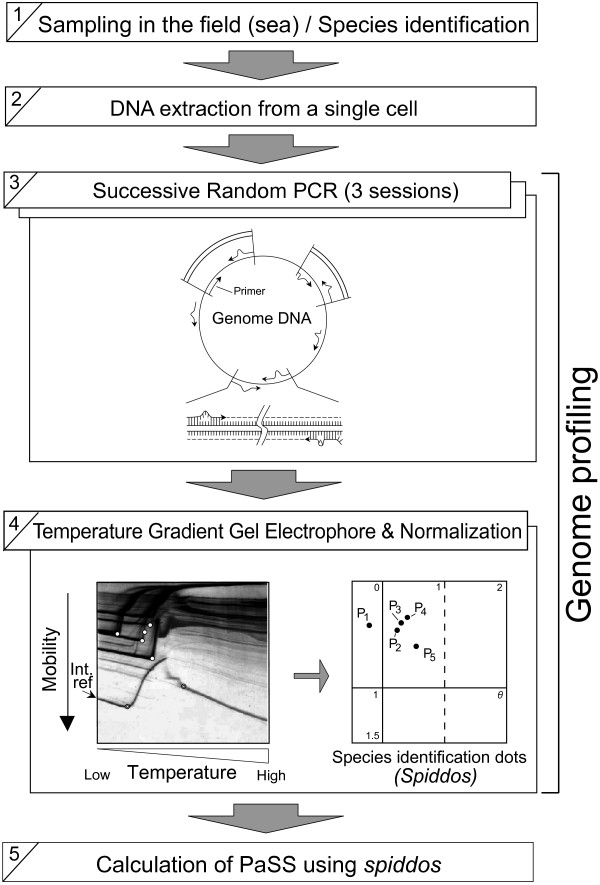
**Reduction of genome information from a single cell into a genome profile**. Successive random PCR, in which the product of one round of random PCR was used as the template for the next session of random PCR, was carried out. Note that primer binding occurs in a mismatch-containing structure in this relaxed mode of PCR (random PCR), thus enabling us to sample DNA fragments from various sites of the genome. In TGGE, DNA fragments layered on the top of a slab gel migrate downward in a horizontal line with a characteristic curvature caused by the temperature gradient. Featuring point(s) of each DNA fragment are assigned and processed to generate *species identification dots *(*spiddos*) with a computer. The PaSS (pattern similarity score) calculation is performed as described in the text.

GP is composed of two major steps: random PCR and temperature gradient gel electrophoresis (TGGE)(Fig. 4). Random PCR was performed using a single primer of dodeca-nucleotides (pfM12, dAGAACGCGCCTG) with the 5'-end Cy3-labeled. This primer sequence has been recommended for general use including the application to animal cells [7]. As one round of PCR amplification of DNA from a single cell does not produce a sufficient amount of DNA, three rounds were employed. The first PCR reaction (50 μl) contained 200 μM dNTPs (N= G,A,T,C), 0.5 μM primer, 10 mM Tris-HCl (pH 9.0), 50 mM KCl, 2.5 mM MgCl_2_, 0.02 unit/μl Taq DNA polymerase (Takara Bio, Shiga, Japan) and a particular amount of template DNA. In the random PCR experiments, special cares were taken as follows ; i) The reaction mixtures just before adding template DNA were UV irradiated. ii) the third session of random PCR was stopped sufficiently before the consumption of the primers so as to keep the products in the double stranded state. Random PCR was carried out with 30 cycles of denaturation (94°C, 30 s), annealing (26°C, 2 min) and extension (47°C, 2 min) using a PTC-100TM PCR machine (MJ Research, Inc., Massachusetts, USA). The second random PCR (50 μl) contained 5 μl of the first PCR products as template and the same concentrations of the other constituents, and the reaction was run for 30 cycles under the same conditions. The third round of random PCR (25 μl) contained 1 μl of the second round products as template. The third round of PCR was stopped after 10 cycles in order to produce as much double-stranded DNA as possible. The DNA samples were subjected to μ-TGGE [11], which adopts a tiny slab gel of 24 × 16 × 1 mm^3 ^for electrophoresis using a temperature-gradient generator, μ-TG (Taitec, Saitama, Japan). In each run of electrophoresis, an internal reference DNA was co-migrated. The 200-bp reference DNA (the 191-bp bacteriophage fd gene VIII, sites 1350~1540 attached to a 9-bp sequence, CTACGTCTC, at the 3'-end) is experimentally determined to have a melting temperature of 60°C under standard conditions. The gel used was 6% acrylamide (acrylamide:bis = 19:1) containing 90 mM Tris-HCl (pH 8.0), 90 mM boric acid, 2 mM EDTA and 8 M urea. The linear temperature gradient was run from 15°C to 60°C. After electrophoresis, DNA bands were detected with a fluorescence imager (Molecular Imager FX, Biorad, Hercules, CA) or by silver staining [11].

### Spiddos and PaSS

Genome profiles obtained by GP are highly informative but difficult to manage due to their complexity. However, this inconvenience was overcome by introducing the featuring points, designated as *spiddos *(species identification dots), which can represent genome profiles compactly [13]. The featuring points, or *spiddos*, correspond to the points where structural transitions of DNA occur, such as double-stranded to single-stranded DNA [19]. *Spiddos *can be used to provide a sufficient amount of information for identifying species [13]. One can easily increase the amount of information about the genome by adding *spiddos *obtained by another GP operated with a different primer. Because the number of possible primers is so large (1.6 × 10^7 ^for dodecanucleotides), many GPs (thus, *spiddos*) can be obtained if necessary. Using *spiddos*, we can define the pattern similarity score (PaSS) of the genomes between two species as follows:

PaSS=1−1n∑i=1n|P→i−P→′i||P→i|+|P→′i|0≦Pass≦1     (1)
 MathType@MTEF@5@5@+=feaafiart1ev1aaatCvAUfKttLearuWrP9MDH5MBPbIqV92AaeXatLxBI9gBaebbnrfifHhDYfgasaacH8akY=wiFfYdH8Gipec8Eeeu0xXdbba9frFj0=OqFfea0dXdd9vqai=hGuQ8kuc9pgc9s8qqaq=dirpe0xb9q8qiLsFr0=vr0=vr0dc8meaabaqaciaacaGaaeqabaqabeGadaaakeaafaqabeqacaaabaGaemiuaaLaemyyaeMaem4uamLaem4uamLaeyypa0JaeGymaeJaeyOeI0YaaSaaaeaacqaIXaqmaeaacqWGUbGBaaWaaabCaeaadaWcaaqaamaaemaabaGafmiuaaLbaSaadaWgaaWcbaGaemyAaKgabeaakiabgkHiTiqbdcfaqzaalyaafaWaaSbaaSqaaiabdMgaPbqabaaakiaawEa7caGLiWoaaeaadaabdaqaaiqbdcfaqzaalaWaaSbaaSqaaiabdMgaPbqabaaakiaawEa7caGLiWoacqGHRaWkdaabdaqaaiqbdcfaqzaalyaafaWaaSbaaSqaaiabdMgaPbqabaaakiaawEa7caGLiWoaaaaaleaacqWGPbqAcqGH9aqpcqaIXaqmaeaacqWGUbGBa0GaeyyeIuoaaOqaaiabicdaWmrtHrhAqLwmaeHbnfgDOvwBHrxAJfgBqLgiXaaceaGae8NzIuIaemiuaaLaemyyaeMaem4CamNaem4CamNae8NzIuIaeGymaedaaiaaxMaacaWLjaWaaeWaaeaacqaIXaqmaiaawIcacaGLPaaaaaa@6A2F@

where P→i
 MathType@MTEF@5@5@+=feaafiart1ev1aaatCvAUfKttLearuWrP9MDH5MBPbIqV92AaeXatLxBI9gBaebbnrfifHhDYfgasaacH8akY=wiFfYdH8Gipec8Eeeu0xXdbba9frFj0=OqFfea0dXdd9vqai=hGuQ8kuc9pgc9s8qqaq=dirpe0xb9q8qiLsFr0=vr0=vr0dc8meaabaqaciaacaGaaeqabaqabeGadaaakeaacuWGqbaugaWcamaaBaaaleaacqWGPbqAaeqaaaaa@2F6E@ and P→′i
 MathType@MTEF@5@5@+=feaafiart1ev1aaatCvAUfKttLearuWrP9MDH5MBPbIqV92AaeXatLxBI9gBaebbnrfifHhDYfgasaacH8akY=wiFfYdH8Gipec8Eeeu0xXdbba9frFj0=OqFfea0dXdd9vqai=hGuQ8kuc9pgc9s8qqaq=dirpe0xb9q8qiLsFr0=vr0=vr0dc8meaabaqaciaacaGaaeqabaqabeGadaaakeaacuWGqbaugaWcgaqbamaaBaaaleaacqWGPbqAaeqaaaaa@2F79@ correspond to the normalized positional vectors (composed of two elements, mobility and temperature) for *spiddos *P_i _and P_i_' collected from two genome profiles (discriminated with or without a prime), respectively, and i denotes the serial number of *spiddos*. A database site has been constructed (*On-web GP *[20]) in order to provide semi-automatic data processing [21]. The PaSS value thus obtained is empirically known to be a good measure to quantify the closeness between two species (or cells) [13].

### PaSS calculation

After calculating the average PaSS value (PaSSA,B¯
 MathType@MTEF@5@5@+=feaafiart1ev1aaatCvAUfKttLearuWrP9MDH5MBPbIqV92AaeXatLxBI9gBaebbnrfifHhDYfgasaacH8akY=wiFfYdH8Gipec8Eeeu0xXdbba9frFj0=OqFfea0dXdd9vqai=hGuQ8kuc9pgc9s8qqaq=dirpe0xb9q8qiLsFr0=vr0=vr0dc8meaabaqaciaacaGaaeqabaqabeGadaaakeaadaqdaaqaaiabdcfaqjabdggaHjabdofatjabdofatnaaBaaaleaacqWGbbqqcqGGSaalcqWGcbGqaeqaaaaaaaa@34B3@), a representative PaSS value for a particular level of taxon T, was obtained over the constituents at a particular level of taxon using the following equations.

PaSSA,B¯=∑jNB∑iNApass(i,j;A,B)NA⋅NB(A≠B)     (2)
 MathType@MTEF@5@5@+=feaafiart1ev1aaatCvAUfKttLearuWrP9MDH5MBPbIqV92AaeXatLxBI9gBaebbnrfifHhDYfgasaacH8akY=wiFfYdH8Gipec8Eeeu0xXdbba9frFj0=OqFfea0dXdd9vqai=hGuQ8kuc9pgc9s8qqaq=dirpe0xb9q8qiLsFr0=vr0=vr0dc8meaabaqaciaacaGaaeqabaqabeGadaaakeaafaqabeqacaaabaWaa0aaaeaacqWGqbaucqWGHbqycqWGtbWucqWGtbWudaWgaaWcbaGaemyqaeKaeiilaWIaemOqaieabeaaaaGccqGH9aqpdaWcaaqaamaaqahabaWaaabCaeaacqWGWbaCcqWGHbqycqWGZbWCcqWGZbWCcqGGOaakcqWGPbqAcqGGSaalcqWGQbGAcqGG7aWocqWGbbqqcqGGSaalcqWGcbGqcqGGPaqkaSqaaiabdMgaPbqaaiabd6eaonaaBaaameaacqWGbbqqaeqaaaqdcqGHris5aaWcbaGaemOAaOgabaGaemOta40aaSbaaWqaaiabdkeacbqabaaaniabggHiLdaakeaacqWGobGtdaWgaaWcbaGaemyqaeeabeaakiabgwSixlabd6eaonaaBaaaleaacqWGcbGqaeqaaaaaaOqaamaabmaabaGaemyqaeKaeyiyIKRaemOqaieacaGLOaGaayzkaaaaaiaaxMaacaWLjaWaaeWaaeaacqaIYaGmaiaawIcacaGLPaaaaaa@60F3@

PaSST=∑Qν∑PνPaSSP,Q¯ν(ν−1)(P≠Q)     (3)
 MathType@MTEF@5@5@+=feaafiart1ev1aaatCvAUfKttLearuWrP9MDH5MBPbIqV92AaeXatLxBI9gBaebbnrfifHhDYfgasaacH8akY=wiFfYdH8Gipec8Eeeu0xXdbba9frFj0=OqFfea0dXdd9vqai=hGuQ8kuc9pgc9s8qqaq=dirpe0xb9q8qiLsFr0=vr0=vr0dc8meaabaqaciaacaGaaeqabaqabeGadaaakeaafaqabeqacaaabaGaemiuaaLaemyyaeMaem4uamLaem4uam1aaSbaaSqaaiabdsfaubqabaGccqGH9aqpdaaeWbqaamaaqahabaWaaSaaaeaadaqdaaqaaiabdcfaqjabdggaHjabdofatjabdofatnaaBaaaleaacqWGqbaucqGGSaalcqWGrbquaeqaaaaaaOqaaGGaciab=17aUnaabmaabaGae8xVd4MaeyOeI0IaeGymaedacaGLOaGaayzkaaaaaaWcbaGaemiuaafabaGae8xVd4ganiabggHiLdaaleaacqWGrbquaeaacqWF9oGBa0GaeyyeIuoaaOqaamaabmaabaGaemiuaaLaeyiyIKRaemyuaefacaGLOaGaayzkaaaaaiaaxMaacaWLjaWaaeWaaeaacqaIZaWmaiaawIcacaGLPaaaaaa@56DA@

**Figure 5:**
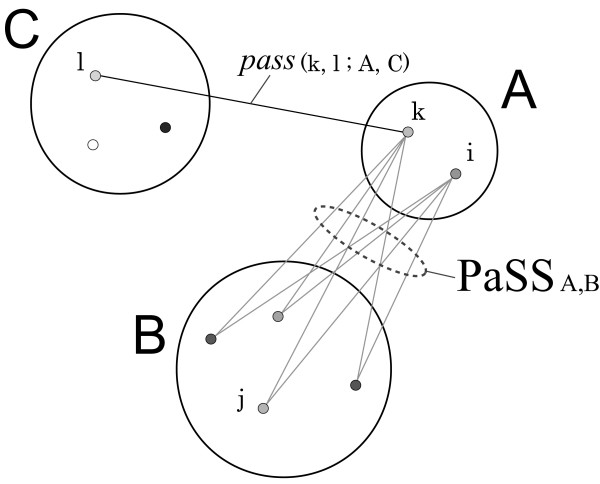
**Schematic drawing of PaSS_T _calculation**. PaSS_A,B _is an average of all combinations of PaSS values: an element belonging to A versus another element belonging to B.

Where *pass (i, j ; A, B) *means the PaSS elementary value between the i-th element of the genome profile of an organism A and j-th element of the genome profile of an organism B ; PaSS_P,Q _means the PaSS value between the *spiddos *for organisms, P and Q ; N_A _and N_B_, stand for the number of *spiddos *belong to A and B, respectively ; and *ν *stands for the number of species (Fig.5).

### Clustering analysis

The group average method [14] was used for the clustering analysis of the genomes of the organisms examined here. With this method, a separate element is integrated into a cluster if the distance is smaller than or equal to a finite value between the element and at least one member of the cluster.

### Sequencing and FASTA analysis

Some of the random PCR products (DNA) were cloned and analyzed by sequencing. In addition, a part of the 18S rDNA was subjected to sequencing. This product was obtained by PCR-amplification using the mixture of random PCR products plus the template genome DNA as a template source and the primers, F (GAGGGCAAGTCTGGT) and R (ACGACGGTATCTGAT), which were designed as in the preceding study [20]. The sequencing was done by adopting the TA cloning method (Invitrogen, Carlsbad, California, USA). The DNA sequencer, DSQ-2000L (Shimadzu, Kyoto, Japan) was used for this purpose. Experimentally obtained DNA sequences were analyzed with FASTA against all of the genomes and genes in the database. DNA segments of more than 55 % similarity were carefully analyzed based on their annotations so as to determine the source of the DNA sequences.

## Competing interests

The author(s) declare that they have no competing interests.

## Authors' contributions

MK performed the experiments and the genome analysis, interpreted the results and prepared the substantial amount of the manuscripts. AM suggested the essence of this study at the beginning of this study, collected the Radiolarian specimens, and contributed to the discussion of this project. KN initiated and organized this study, helped interpreting the results and also helped drafting the manuscripts. Both of AM and KN contributed to supporting this study financially.

## Figures and Tables

**Table 1:** Organisms used in this study and their taxonomic classification

Code^†^	Species	Taxon*			Source^§ ^(ID^‡^)
A_1_-1	*Didymocyrtis tetrathalamus*	Pc ^#^	Rh	Pro	1 (3354)
A_1_-2	*Didymocyrtis tetrathalamus*	Pc ^#^	Rh	Pro	1 (3356)
A_2_	*Dictyocoryne profunda*	Pc ^#^	Rh	Pro	2 (3176)
A_3_-1	*Dictyocoryne truncatum*	Pc ^#^	Rh	Pro	2 (3175)
A_3_-2	*Dictyocoryne truncatum*	Pc ^#^	Rh	Pro	2 (3174)
A_3_-3	*Dictyocoryne truncatum*	Pc ^#^	Rh	Pro	1 (3360)
A_4_	*Hymeniastrum euclidis*	Pc ^#^	Rh	Pro	3 (3032)
A_5_	*Eucyrtidium hexasticum*	Pc ^#^	Rh	Pro	3 (3039)
B	A species belonging to Acantharia^$^	Acantharia^#^	Rh	Pro	4 (3153)
C	A species belonging to Foraminifera^$^	Foraminifera	Rh	Pro	4 (3147)
D-1	A species belonging to Phaeodaria^$^	Ph^#^	Rh	Pro	4 (3150)
D-2	A species belonging to Phaeodaria^$^	Ph^#^	Rh	Pro	4 (3151)
E	A species belonging to Diatomophyceae^$^	Heterokontophyta	→|	Pro	4 (3148)
F	*Saccharomyces cerevisiae*	Fungi	→	→|	5 (-)
G	*Escherichia coli *(S26)	Ba	→	→|	5 (-)
H	*Bacillus subtilis*	Ba	→	→|	5 (-)

^† ^Alphabetical codes stand for a taxon of organism. The subdivision of a taxon is shown by a suffix. Such clone is called by the additional number.

* Pc: Polycystinea, Ph: Phaeodaria, Ba: Bacteria, Rh: Rhizaria, Pro: Protista.

^§ ^Each number represents the sampling location and date; **1**. off-shore of Okinawa island, Japan/May 2005. **2**. off-shore of Okinawa island, Japan/Nov. 2004. **3**. off-shore of Okinawa island, Japan/Dec. 2003. **4**. Sado island, Japan/Jul. 2004. **5**. *Saccharomyces cerevisiae *was commercially obtained from Nisshin Flour Milling Co.,Ltd. *Escherichia coli *(S26) was obtained from the Saitama Institute of Public Health (Saitama, Japan). Genomic DNA of *Bacillus subtilis *was a gift from Dr. Itaya (Mitsubishi Institute of Life Sciences, Japan). F-H used the data registered as reference data.

^‡ ^Experimental serial no.

^# ^Haeckel's Radiolaria.

^$ ^Nomenclature at the taxonomical level of class.

**Table 2:** Distance between the constituent elements at each taxon measured with PaSS.

Taxa*	average PaSS
Phylum/Devision	0.550 (-)
Class	0.656 (± 0.034)
Order	0.761 (± 0.090)
Family	0.782 (± 0.056)
Genus	0.822 (± 0.034)

* Organisms examined belong to Proteobacteria, Gram-positive low-G+C group, Basidiomycetes, Bryopsida, Monocotyle-donopsidae, Dicotyledoneae, Mammalia, Aves at the taxon level by class. The primers used were oligonucleotides : dAGAACGCGCCTG (pfM12) and dCAGGGCGCGTAC (pfM19)

^# ^The average PaSS value for non-related pairs of organisms had been statistically obtained to be 0.435 ± 0.091 (Ref. 13).
